# Case Report: Intravascular ultrasound-guided management of acute myocardial infarction in a rare coronary artery case with multiple ostia and varied lesions

**DOI:** 10.3389/fcvm.2024.1374240

**Published:** 2024-05-10

**Authors:** Huabin He, Qin Ao, Jianhai Chen, Jun Zhang, Xiangyang Yuan

**Affiliations:** ^1^ Department of Cardiovascular Medicine, Jiujiang No. 1 People’s Hospital, Jiujiang, China; ^2^ Department of Cardiovascular Medicine, The Second Affiliated Hospital, Jiangxi Medical College, Nanchang University, Nanchang, China

**Keywords:** coronary artery anomalies (CAAs), anomalous origin of the coronary artery (AOCA), acute myocardial infarction, intravascular ultrasound (IVUS), the right circumflex artery (RCX), coronary artery intervention treatment

## Abstract

Anomalous origin of the coronary artery (AOCA) in coronary arteries represent a rare congenital variation, especially when three or more openings coexist, accompanied by conditions such as myocardial infarction, acute heart failure, and severe stenosis in three vessels, making it even rarer. This study reports a rare case of a patient admitted for the first time with acute myocardial infarction. Coronary angiography revealed four openings, along with the aforementioned rare conditions. Guided by intravascular ultrasound (IVUS), treatments were administered for different lesions in various vessels. IVUS confirmed a rare case with a 1 mm extremely short left main coronary artery and three openings. The two-year follow-up results for this patient are deemed satisfactory, indicating a favorable prognosis.

## Introduction

Anomalous Origin of the Coronary Artery (AOCA) are present at birth, and they are relatively uncommon findings in coronary angiography (1), particularly when three or more openings are present, accompanied by myocardial infarction, acute heart failure, and severe stenosis in three vessels. This study reports a case of an elderly male admitted with acute myocardial infarction, where coronary angiography revealed four openings. Subsequent intravascular ultrasound (IVUS) confirmed this as a rare case of three openings combined with a 1 mm extremely short left main coronary artery. Severe stenosis was observed in all three vessels, leading to appropriate coronary intervention treatment. Two years of follow-up revealed favorable clinical treatment outcomes. This case aims to contribute to the understanding of coronary artery anomalies, particularly anomalous origin, providing further insights for healthcare professionals and supplementing the existing literature on coronary artery anomalies.

## Case report

**Figure 1:**
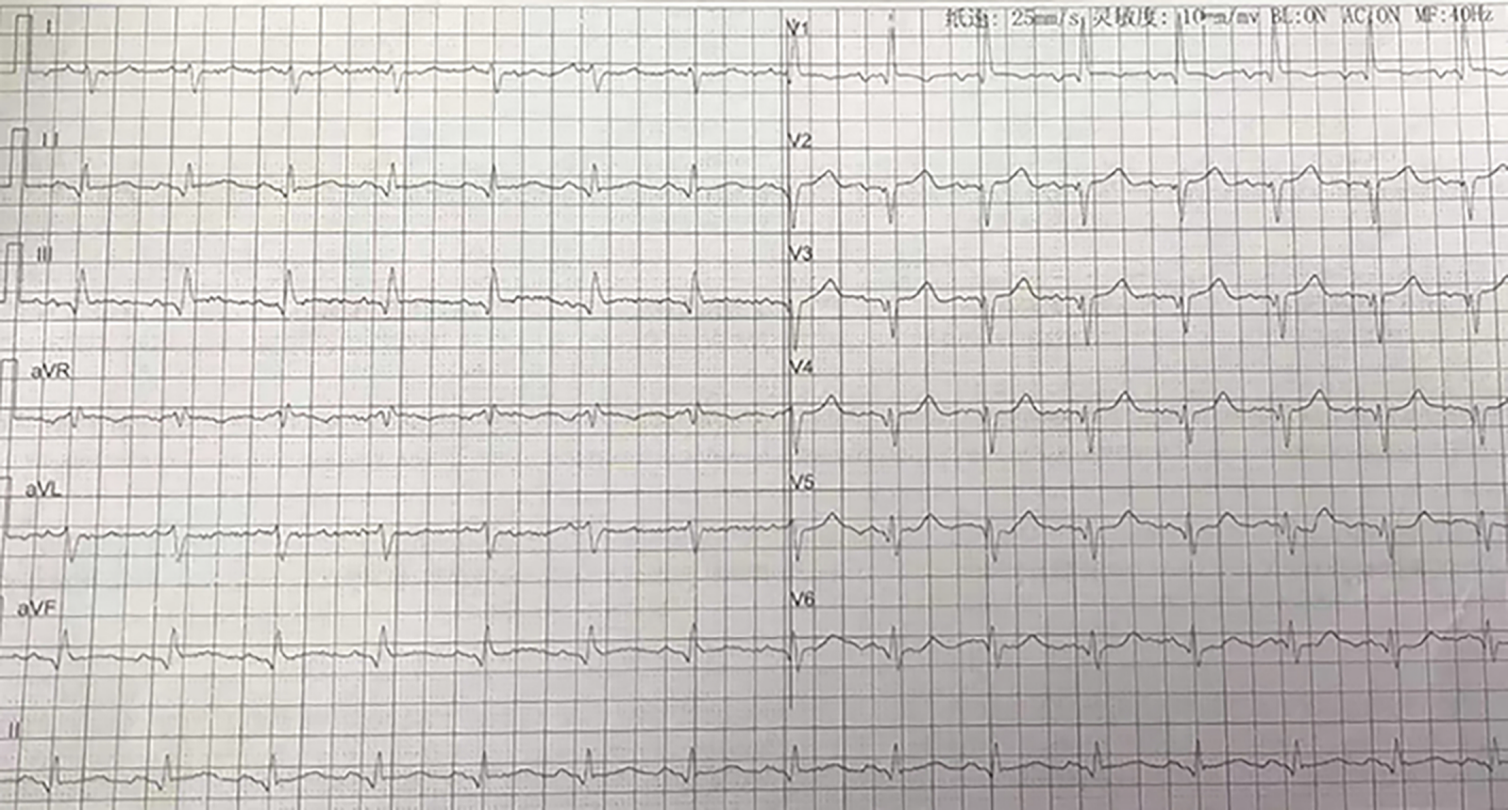
ECG in the emergency department demonstrating displayed sinus rhythm, incomplete right bundle branch block, and ST-segment elevation in leads II, III, AVF, with poor R-wave progression in leads V1–V6. ECG, electrocardiography.

**Figure 2:**
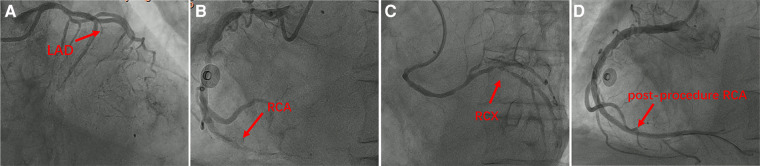
The initial emergency coronary angiography during the first hospitalization: (**A**) left coronary angiography revealed complete occlusion of the proximal left anterior descending artery (LAD) after the origin of the diagonal branch, with no residual segment observed. The left circumflex artery appeared small, and no significant stenosis was observed. (**B**) Right coronary angiography showed diffuse and severe lesions throughout the entire length of the right coronary artery (RCA) with moderate to severe calcification. The distal segment presented total occlusion with a high thrombus burden, and a suspicious vessel shadow was noted below the ostium of the RCA. (**C**) A rare finding was observed where a branch similar to the left circumflex artery arose approximately 1–2 cm below the origin of the right coronary artery, also originating from the right sinus. There were scattered plaques in the proximal and mid segments of this branch, with an eccentric stenosis of approximately 90% at the bifurcation. We refer to this vessel as the right circumflex artery (RCX). (**D**) Right coronary artery emergency PCI images indicated resolution of vessel occlusion, absence of residual stenosis, and restoration of blood flow to TIMI 3 levels.

**Figure 3:**
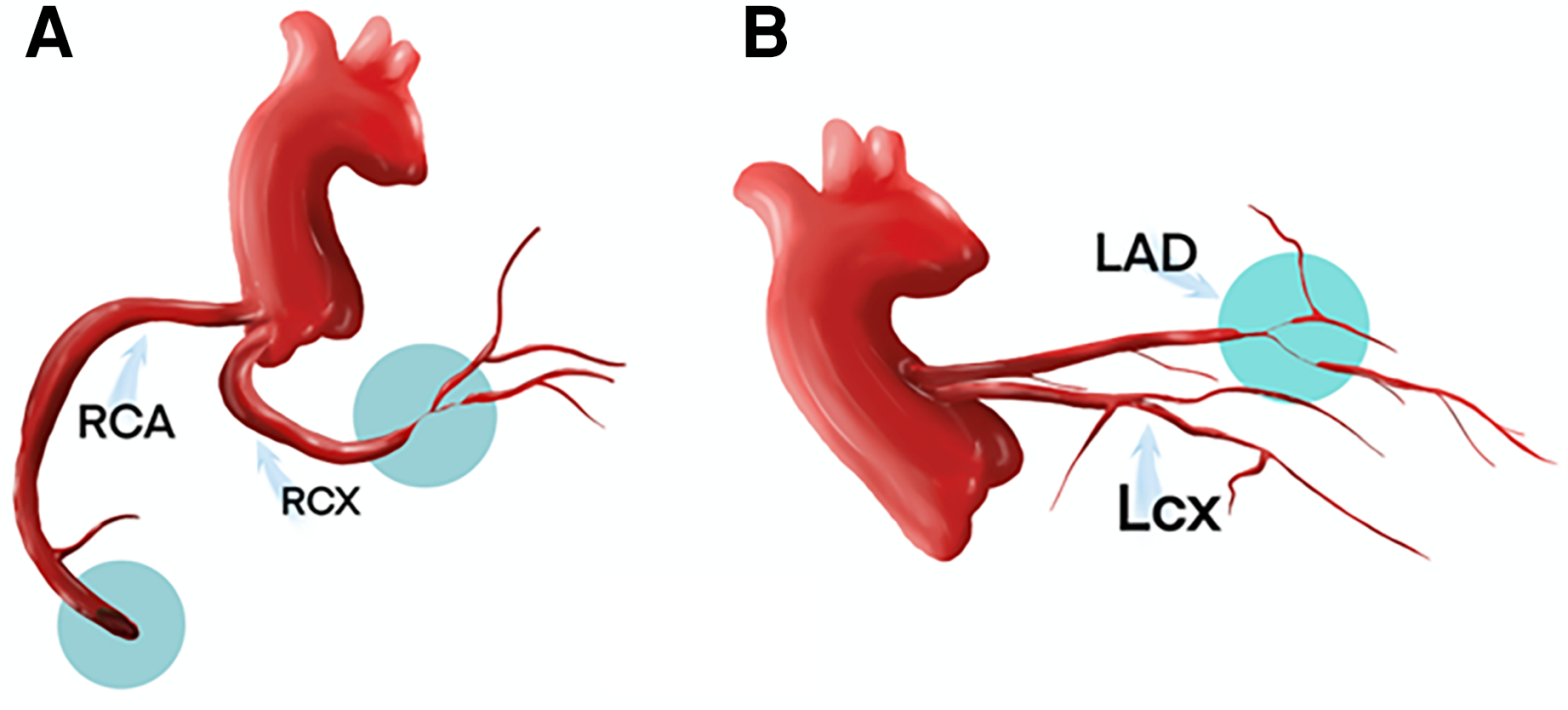
The coronary artery anomalies and sites of obstructive lesions are described in the central illustration.

An 85-year-old male patient was urgently admitted to the emergency department due to “recurrent chest tightness and dyspnea for the past 20 days, with worsening symptoms in the last 11 h.” The patient had a medical history of chronic obstructive pulmonary disease and chronic renal insufficiency. There was no documented history of diabetes, hyperlipidemia, hypertension, familial coronary artery disease, or smoking. Upon admission, the physical examination revealed a blood pressure of 125/84 mmHg, a heart rate of 87 beats/min, and prominent dry and wet rales in both lungs. Bedside cardiac markers showed elevated levels of cTNI (6.79 ng/ml), CK-MB (9.96 ng/ml), and myo (85.8 ng/ml). Bedside ultrasound revealed bilateral pleural effusion (92 mm on the right, 71 mm on the left) and a small amount of pericardial effusion. Additionally, thinning of the left ventricular posterior wall was noted. The electrocardiogram (ECG) (Figure 1) displayed sinus rhythm, incomplete right bundle branch block, and mild ST-segment elevation in leads II, III, AVF, with poor R-wave progression in leads V1–V6. The initial diagnosis upon emergency admission considered acute myocardial infarction with concomitant acute heart failure. Upon admission, emergency coronary angiography was immediately performed using a 5F angiographic catheter (Figures 2A–C), revealing a tri-ostial anomaly in the coronary arteries. The coronary artery anomalies and sites of obstructive lesions are described in the Central Illustration (Figure 3). The mid to distal segment of the left anterior descending artery (LAD) was completely occluded with chronic total occlusion, and the ostium was unclear. The left circumflex artery (LCX) appeared small without significant stenosis. The right coronary artery showed diffuse and severe lesions with moderate to severe calcification throughout its entire course, and the distal segment exhibited total occlusion, indicative of a lesion with a high thrombus burden. The right circumflex artery (RCX) was large, originating from the right sinus, with scattered plaques in the proximal and mid segments, and eccentric stenosis of approximately 90% at the bifurcation, achieving TIMI 3 flow. Then, coronary artery bypass surgery (CABG) was recommended for the patient, but he and his family rejected this recommendation, opting instead for repercutaneous coronary intervention (PCI) treatment. A 6F XBRCA guide catheter was then advanced under fluoroscopic guidance to the ostium of the right coronary artery (RCA). Following thrombus aspiration, pre-dilation with a 2.5 mm × 20 mm balloon, intracoronary drug injection, and other pre-treatments, drug-eluting stents (DES) were sequentially implanted in the left posterior descending artery from the near segment to the opening of the right coronary artery using stents sized 2.75 mm × 33 mm, 3.0 mm × 29 mm, 3.5 mm × 28 mm, and 4.0 mm × 29 mm. Post-dilation was performed using 3.0 mm × 12 mm, 3.5 mm × 12 mm, and 4.0 mm × 12 mm non-compliant balloons at pressures of 16 atm–26 atm. The final angiography showed satisfactory stent apposition with no dissection, and TIMI 3 flow was successfully restored (Figure 2D). Preoperative laboratory results indicated NT-ProBNP: 8,952 pg/ml, creatinine: 163 umol/L. Echocardiography revealed a left ventricular end-diastolic diameter (LVDd) of 55.8 mm and left ventricular ejection fraction (LVEF) of 41.7%. During hospitalization, the patient developed paroxysmal atrial fibrillation and atrial flutter. Following discharge, the patient received secondary prevention medications for coronary heart disease and anticoagulation, with plans for staged interventions on the RCX and LAD scheduled for one month later.

**Figure 4:**
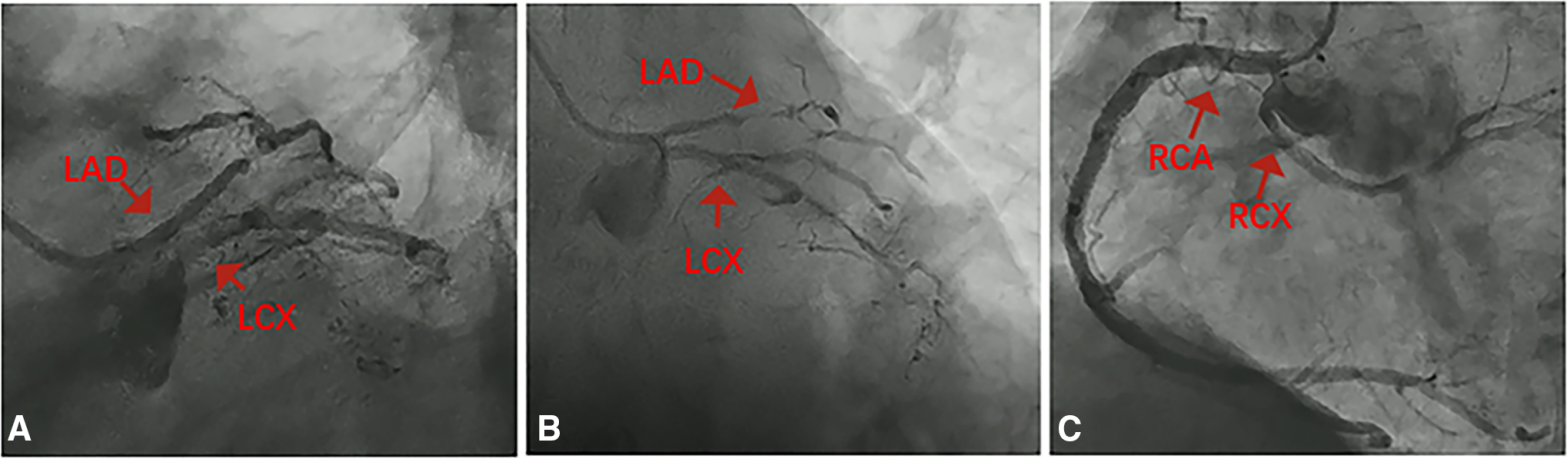
The second hospitalization coronary angiography: (**A,B**) the left anterior descending artery and left circumflex artery also exhibit dual openings. (**C**) Further confirmation reveals dual openings of the right coronary artery and right circumflex artery at the right coronary sinus.

**Figure 5:**
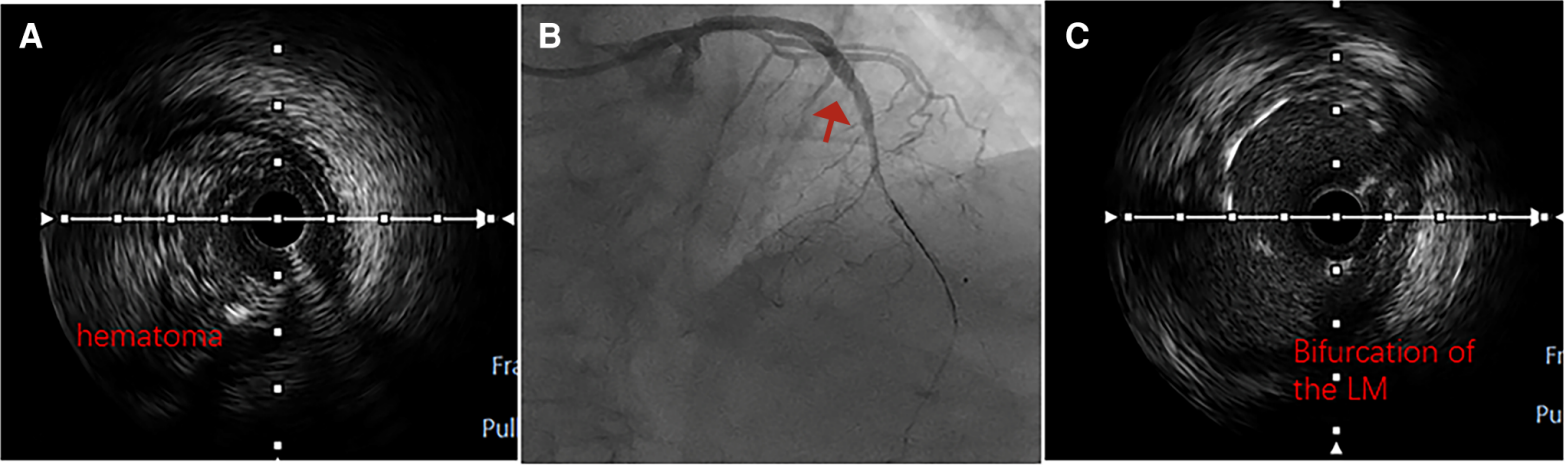
Intraoperative and postoperative intravascular ultrasound (IVUS) images, as well as coronary angiography: (**A**) after pre-dilation, IVUS revealed a large intramural hematoma. (**B**) Final angiography of the left anterior descending artery showed satisfactory apposition of the stent to the vessel wall, with no signs of dissection, and forward blood flow rated as TIMI3. (**C**) Intravascular ultrasound demonstrated that the stent in the left anterior descending artery extended into the left main trunk, further confirming the presence of a common origin of the left anterior descending and left circumflex arteries with the left main coronary artery measuring approximately 1 mm in length.

One month later, a subsequent coronary angiography (Figures 4A,B) was conducted using a 5F angiographic catheter, revealing a coronary artery quadrifurcation anomaly. Imaging suggested that the left anterior descending artery (LAD) and left circumflex artery (LCX) did not share a common opening. The mid-segment of the LAD was subtotally occluded, and the LCX was small without significant stenosis, resulting in TIMI 0 flow in the LAD. Firstly, a 6F XBRCA guide catheter was then advanced to the ostium of the RCX. Intervention was performed on the large bifurcation lesion in the RCX using a 2.0 mm × 20 mm pre-dilation balloon, a 2.75 mm × 6 mm cutting balloon, and a 2.5 mm × 12 mm post-dilation balloon. Angiography after the procedure (Figure 4C) showed no residual stenosis or dissection, and forward blood flow was successfully restored with TIMI 3. Secondly, 7F EBU 3.5 guide catheter was then advanced under fluoroscopic guidance to the left main coronary artery. Intravascular ultrasound (IVUS) examination was performed on the mid-segment to the opening of the left anterior descending artery. IVUS revealed a short left main stem, technically a tri-ostial anomaly. The mid to near segments showed diffuse fibrocalcific plaques with severe stenosis, and the mid-segment showed calcified nodules. The minimum lumen area was less than 2.0 mm^2^, with plaque burden of 57% at the bifurcation. Pre-dilation with 1.5 mm × 20 mm and 2.0 mm × 20 mm balloons was performed, and angiography after forward blood flow was successfully restored in the left anterior descending artery. During the procedure, the guidewire dislodged but was successfully re-advanced using an XT-R guidewire to reach the distal left anterior descending artery. IVUS examination (Figure 5A) showed a hematoma in the near segment. A drug-eluting stent (DES) measuring 2.5 mm × 18 mm and 3.0 mm × 33 mm was sequentially implanted in the mid-segment to the opening of the left anterior descending artery, followed by post-dilation with a 3.0 mm × 12 mm balloon. Postoperative coronary angiography (Figure 5B) and IVUS (Figure 5C) confirmed satisfactory stent expansion with no dissection, and the stent protruded into the left main stem by approximately 1 mm. Six months after discharge, the patient's 6 min walk test exceeded 425 m, and there were no symptoms of chest discomfort. Repeat echocardiography showed LVDd: 51 mm and LVEF: 58%. The patient's condition significantly improved, and a telephone follow-up 2 years after discharge indicated an unrestricted 6 min walk test without any discomfort symptoms.

## Discussion

Coronary artery anomalies (CAAs) refer to a group of congenital coronary artery diseases, which can generally be divided into anomalies in the origin of the coronary artery, anomalies in the termination of the coronary artery, and anomalies in the internal anatomical structure of the coronary artery (2). Among these, anomalies of coronary artery origin (AOCA) are a subtype of coronary artery variations. According to current literature, their incidence rate is about 0.5%–1.3%, often asymptomatic or with hidden symptoms, making it an extremely rare disease (3–5). The main types of variations include: the left and right coronary arteries opening into a single coronary sinus; the right coronary artery originating from a non-coronary sinus; the left circumflex originating from the right coronary sinus; the anterior descending branch and left circumflex not having a left main stem, opening independently into the left coronary sinus; coronary artery originating from the pulmonary artery; and a single coronary artery (6). This article reports a case that presents a combination of conditions described above, which is an extremely rare occurrence in terms of both its characteristics and the course of diagnosis and treatment. From a variation perspective, there is a presence of a double helix, with a large helical branch opening into the right sinus and a corresponding smaller left helical branch; the anterior descending and left helical branches appear similar to a double opening on angiography, that is, a case of four openings. However, intravascular ultrasound (IVUS) confirmed it as a case of three openings combined with an extremely short left main trunk, which has not been reported in domestic or international literature. In terms of anatomical and physiological mechanisms, the left helical branch originates from the right coronary sinus, defined here as the right helical branch, which supplies blood to the area normally served by the left helical branch while also overlapping with the area supplied by the right coronary artery. The culprit vessel in this case of acute myocardial infarction was the occlusion of the right coronary artery, but the substantial right helical branch provided some compensation to myocardial blood supply, preventing common risks such as cardiogenic shock or sudden cardiac death. This variation could theoretically be beneficial, but unfortunately, there is a lack of coronary CT angiography and other imaging data to corroborate this.

From the current research perspective on anomalous origin of the coronary artery (AOCA), there is ongoing debate about whether AOCA patients are more susceptible to coronary atherosclerosis. Some experts argue that AOCA, often asymptomatic, has no impact on obstructive atherosclerotic plaques in the coronary arteries, given its lack of clinical symptoms (7). However, many scholars hold opposing views, contending that the abnormal course of vessels significantly influences blood flow shear forces, inevitably leading to a notable increase in the incidence of atherosclerotic plaques (8–10). Current research emphasizes blood flow shear force as the primary mechanism leading to the formation of obstructive atherosclerotic plaques, with high shear force areas predominantly located in the narrowed proximal segments, making them prone to plaque rupture (11). Analyzing the characteristics of the lesion reported in this case, coronary angiography reveals a quadrifurcation anomaly, concomitant with three severe stenoses: complete occlusion (100%), near-total occlusion (99%), and severe narrowing (90%). Additionally, there is a coexistence of high thrombus burden, bifurcation lesions, and chronic occlusive lesions, marking this as an extremely high-risk and complex coronary intervention case. This further underscores the substantial association between the abnormal course and variation of vessels and the development of obstructive plaques in the coronary arteries. However, this association lacks confirmation from large-scale clinical studies and requires further exploration through additional data.

Percutaneous coronary intervention is a common treatment for obstructive lesions in AOCA. This particular case involves intervention strategies for multiple lesions on the basis of anatomical variations, including: treatment protocols for thrombotic lesions in acute myocardial infarction; strategy selection and standardized implantation of drug-eluting balloons for bifurcation lesions; and intervention treatment for chronic occlusive coronary lesions. IVUS confirmed an extremely short left main trunk, which is difficult to detect with the naked eye, necessitating pre-placement of a balloon for protection. The massive hematoma image provided by IVUS during surgery guided the pressure for stent release and post-dilation.

Current research suggests that the risk of severe acute coronary syndrome associated with AOCA is more pronounced in individuals aged 12–35 years, and vascular reconstruction is recommended. Moreover, the risk of morbidity and mortality tends to decrease with advancing age (12, 13). However, the case presented in this article involves an 84-year-old patient with acute myocardial infarction combined with acute left heart failure, a condition that is uncommon both domestically and internationally. Rapid blood flow reconstruction for acute myocardial infarction was initially performed to salvage more myocardial cells and correct complications such as heart failure and arrhythmias. Subsequently, precise intervention treatment for other severe stenoses was conducted under the guidance of intravascular ultrasound. The results were confirmed to be satisfactory through angiographic and functional examinations. Postoperatively, the patient received standardized medication treatment, leading to favorable short-term and long-term clinical outcomes.

The combination of multiple ostia and varied lesions with acute myocardial infarction and multi-vessel disease is an extremely rare case. Through staged interventions addressing individual vessel lesions and conducting systematic follow-ups, there has been a substantial enhancement in the early recognition of coronary artery variations and the refinement of treatment strategies among a broad spectrum of healthcare professionals.

## Data Availability

The raw data supporting the conclusions of this article will be made available by the authors, without undue reservation.

## References

[1] Niu Q Zhao Y Li H Wang Q Zhu H Bi C Case report: treatment of a patient with STEMI and cardiogenic shock caused by RCA originating from LAD. Front Cardiovasc Med. (2022) 9:1036274. 10.3389/fcvm.2022.1036274 36704452 PMC9871765

[2] Lau WR Lee PT Koh CH. Coronary artery anomalies—state of the art review. Curr Probl Cardiol. (2023) 48:101935. 10.1016/j.cpcardiol.2023.101935 37433414

[3] Zhang LJ Yang GF Huang W Zhou CS Chen P Lu GM. Incidence of anomalous origin of coronary artery in 1879 Chinese adults on dual-source CT angiography. Neth Heart J. (2010) 18(10):466–70. 10.1007/BF03091817 20978590 PMC2954298

[4] Villines TC Devine PJ Cheezum MK Gibbs B Feuerstein IM Welch TS. Incidence of anomalous coronary artery origins in 577 consecutive adults undergoing cardiac CT angiography. Int J Cardiol. (2010) 145(3):525–6. 10.1016/j.ijcard.2010.04.059 20451266

[5] Jim MH Siu CW Ho HH Miu R Lam YM Lam L Anomalous origin of right coronary artery from the left coronary sinus: incidence, characteristics, and a systematic approach for rapid diagnosis. J Interv Cardiol. (2005) 18(2):101–6. 10.1111/j.1540-8183.2005.04046.x 15882155

[6] Grani C Padalino MA. Editorial: coronary artery anomalies: a 2020 review. Front Cardiovasc Med. (2022) 9:776951. 10.3389/fcvm.2022.776951 35224033 PMC8866643

[7] Chandra S Singh V Nehra M Agarwal D Singh N. ST-segment elevation in non-atherosclerotic coronaries: a brief overview. Intern Emerg Med. (2011) 6(2):129–39. 10.1007/s11739-010-0491-5 21153605

[8] Parker JA Peivandi AA Bitschnau S Kayhan N Vahl CF. Anomalous origin and retropulmonary course of an atherosclerotic stenosed left circumflex coronary artery. Int J Vasc Med. (2010) 2010:490858. 10.1155/2010/490858 21151519 PMC2989650

[9] Silva J Costa M Mota P Leitão-Marques AM. Myocardial infarction with anomalous coronary anatomy. Rev Port Cardiol. (2009) 28(2):201–5. PMID: 19438155 19438155

[10] Balaguer-Malfagon JR Estornell-Erill J Vilar-Herrero JV Pomar-Domingo F Federico-Zaragoza P Paya-Serrano R. Anomalous left coronary artery from the right sinus of Valsalva associated with coronary atheromatosis. Rev Esp Cardiol. (2005) 58(11):1351–4. 10.1157/13080964 16324589

[11] Lovett JK Rothwell PM. Site of carotid plaque ulceration in relation to direction of blood flow: an angiographic and pathological study. Cerebrovasc Dis. (2003) 16(4):369–75. 10.1159/000072559 13130178

[12] Basso C Maron BJ Corrado D Thiene G. Clinical profile of congenital coronary artery anomalies with origin from the wrong aortic sinus leading to sudden death in young competitive athletes. J Am Coll Cardiol. (2000) 35(6):1493–501. 10.1016/S0735-1097(00)00566-0 10807452

[13] Angelini P. Coronary artery anomalies—current clinical issues: definitions, classification, incidence, clinical relevance, and treatment guidelines. Tex Heart Inst J. (2002) 29(4):271–8. PMID: 12484611 12484611 PMC140289

